# Complete Genome Sequence of *Magnetospirillum* sp. Strain XM-1, Isolated from the Xi’an City Moat, China

**DOI:** 10.1128/genomeA.01171-16

**Published:** 2016-10-20

**Authors:** Yinzhao Wang, Tongwei Zhang, Wei Lin, Bingfang Zhang, Yao Cai, Caiyun Yang, Jinhua Li, Huangtao Xu, Yongxin Pan

**Affiliations:** a PGL, Key Laboratory of Earth and Planetary Physics, Institute of Geology and Geophysics, Chinese Academy of Sciences, Beijing, China; b France-China Bio-Mineralization and Nano-Structures Laboratory, Institute of Geology and Geophysics, Chinese Academy of Sciences, Beijing, China; c University of Chinese Academy of Sciences, Beijing, China

## Abstract

The magnetotactic bacterium *Magnetospirillum* sp. strain XM-1 was recently isolated from the Xi’an City moat, China. It belongs to the *Rhodospirillaceae* family in the *Alphaproteobacteria* class. Here, we report the complete genome sequence of XM-1. The genome contains a single circular chromosome of 4,825,187 bp and a plasmid of 167,290 bp.

## GENOME ANNOUNCEMENT

Magnetotactic bacteria are able to synthesize intracellular nanosized magnetic crystals (Fe_3_O_4_/Fe_3_S_4_) usually arranged into a chain structure, known as magnetosomes, that help the cells navigate along the geomagnetic field in aquatic environments (1, 2). *Magnetospirillum* sp. strain XM-1, belonging to the *Alphaproteobacteria* was isolated from the eutrophic city moat in Xi’an, China (3), and synthesizes about 10 magnetite magnetosomes per cell (4). 

The complete genome of *Magnetospirillum* sp. strain XM-1 was sequenced using Illumina HiSeq 2000 sequencing platforms (BGI, China). Short-insert (~500-bp) and long-insert (~6,000-bp) size DNA libraries were constructed, and a total of 121,032,268 reads were obtained, corresponding to 1,089 Mb of sequence data. *De novo* and reference-based assemblies were performed by SOAP*denovo* version 2.04 (5). Genome of *Magnetospirillum magneticum* AMB-1 (6) was used as a reference strain. For highly complex region, PCRs were conducted to fill all the gaps.

The complete genome sequence of strain XM-1 comprised one circular chromosome of 4,825,187 bp and one plasmid of 167,290 bp. The G+C contents of the chromosome and plasmid are 65.64% and 66.48%, respectively. Gene annotation and analysis were performed using the MaGe Genoscope Web-based system (7). All predicted open reading frames were manually checked. The chromosome contains 4,766 coding sequences (CDSs), 53 tRNAs, two sets of rRNA operons, and 11 miscellaneous RNAs. The average CDS length is 940 bp. Of 4,766 CDSs, 74.26% could be classified as at least one category of the Clusters of Orthologous Groups (COG), in which genes related to signal transduction mechanisms (10.93%) account for dominant COG categories. Inorganic ion transport and metabolism correspond to 5.6% of the gene products in COGs. The plasmid contains 186 CDSs, and their average length is 817 bp. Compared with the chromosome, only 38.17% of plasmid CDSs could be classified as COG groups. Genes associated with replication, recombination, and repair (8.6%) rank first in COG groups in plasmids while making up only 5.7% in the chromosome.

Magnetosome formation in magnetotactic bacteria is controlled by a cluster of conserved genes (8). In *Magnetospirillum* sp. strain XM-1, we found that these essential genes are organized in *mamGFDC*, *mms6*, *mamAB*, and *mamXY* clusters in an approximately 100-kb region. A total of 39 genes are predicted related to magnetosome synthesis, including *fur*, *feoAB*, and *ftsZ*-like genes, and 24 genes were annotated as transposases or mobile elements (9).

Strain XM-1 possesses a megaplasmid containing three clusters of heavy metal efflux genes, one *czcD* gene, and *merR*-like heavy metal regulation genes. These outer membrane heavy metal efflux genes can help bacteria export the toxic heavy metal (10). Five DNA methylase-related genes, one putative type II restriction enzyme gene, a resolvase domain-containing protein gene, and a putative DNA topoisomerase are present in the plasmid, which may help bacteria avoid foreign DNA invasion (11, 12).

### Accession number(s).

The complete genome sequence of *Magnetospirillum* sp. strain XM-1 has been deposited at DDBJ/ENA/GenBank under the accession numbers LN997848 and LN997849.
